# Health Related Quality of Life T-14 Outcomes for Pediatric Bizact Tonsillectomy

**DOI:** 10.3390/medicina57050480

**Published:** 2021-05-12

**Authors:** Lia Stepan, Lucy Huang, Julie Huynh, Phillip Xie, Charmaine M. Woods, Eng H. Ooi

**Affiliations:** 1 Department of Otolaryngology, Head and Neck Surgery, Flinders Medical Centre, Adelaide 5025, Australia; lucy.huang.tw@gmail.com (L.H.); huyn0078@flinders.edu.au (J.H.); philip.y.xie@gmail.com (P.X.); Charmaine.woods@flinders.edu.au (C.M.W.); eooi.entsurgery@gmail.com (E.H.O.); 2 Flinders Health and Medical Research Institute, College of Medicine and Public Heath, Flinders University, Adelaide 5025, Australia

**Keywords:** tonsillectomy, child, tonsillitis, postoperative hemorrhage, quality of life

## Abstract

*Objectives*: The objective of this study was to assess the T-14 outcomes of the Bizact^TM^ device for tonsillectomy in a pediatric population. *Methods*: A case series chart review was undertaken at a public tertiary teaching hospital and private otolaryngology practice, with data collected from pediatric patients who underwent a Bizact^TM^ tonsillectomy between July 2016 and October 2019 for any indication, whose parents consented to completing the T-14 questionnaire providing a parental perspective of the child’s quality of life. Primary outcomes were T-14 scores recorded preoperatively and 6 weeks post-operatively. Secondary outcome measures were postoperative complications, including hemorrhage and readmission. *Results:* 146 patients were identified. There was a significant improvement in T-14 scores from a median of 24 (Interquartile range (IQR) 18–33) prior to surgery to 2 (IQR 0–4) at 6 weeks postoperatively (*p* < 0.001). The post-tonsillectomy hemorrhage rate was 6.1% (9/146 participants). *Conclusions*: Pediatric Bizact^TM^ tonsillectomy is effective in treating common indications for pediatric tonsillectomy, reflected by improved parent-reported health-related quality of life T-14 scores postoperatively.

## 1. Introduction

Bizact^TM^ is a new tonsillectomy device using Valley Lab adjustable bipolar energy to cauterize, seal vessels and divide tissue (Medtronic, Minneapolis, MN, USA). A pilot study presented the findings of Bizact^TM^ tonsillectomies in 186 patients regarding safety and efficacy [1]. With any intervention, technical outcomes, performance-based outcomes, and patient-reported outcomes are important to measure. From a technical perspective, the Bizact^TM^ device has been shown to be user-friendly and easy to set up, and is used to perform a tonsillectomy with an extra-capsular dissection technique [1]. Performance-based outcomes consider intraoperative and postoperative complications, including hospital readmission. Krishnan et al. found in 186 patients (85 adults and 101 pediatric patients) the technique was associated with minimal intraoperative blood loss (97.3% of patients having less than 10 mL blood loss), no primary hemorrhages, and a secondary hemorrhage rate of 4.3% [1]. This hemorrhage rate is comparable with other techniques in the literature [2]. Krishnan’s study comprised a mixture of pediatric and adult patients, with the majority of tonsillectomies performed by two senior consultants.

There are currently no studies in the literature which focus on patient or parental reported health-related quality of life (QOL) outcomes following Bizact^TM^ tonsillectomy in a pediatric population. The patient-reported outcome tools validated for pediatric tonsillectomy are mostly parentally reported. Kao et al. (2017) presented a scoping review of validated pediatric QOL tools for tonsillectomy, and found the only validated, disease-specific tool to cover both infective and obstructive indications for tonsillectomy was the T-14 questionnaire [3]. The T-14 questionnaire was previously used in an Australian population, where T-14 scores were gathered at the preoperative appointment, and at 6 weeks postoperatively [4]. An improvement in the T-14 score 6 weeks postoperatively indicates that the tonsil-related disease burden was decreased. Changes in the T-14 score can occur with both conservative or surgical management. However, surgical management results in an improvement in the T-14 score of greater magnitude and over a shorter time frame than occurs with conservative management [5].

The current study aims to assess parental reported health-related quality of life outcomes specific to Bizact^TM^ tonsillectomy, using T-14 scores, in a pediatric population.

## 2. Materials and Methods

### 2.1. Ethical Considerations

Ethics approval was provided by the Southern Adelaide Clinical Human Research Ethics Committee (project code 123.17, HREC reference: HREC/17/SAC/206, approved 17 October 2017).

### 2.2. Study Design

A case series by chart review of pediatric patients, from 1 to 16 years, who underwent a Bizact^TM^ tonsillectomy at Flinders Medical Centre in Adelaide, South Australia (FMC, a public tertiary teaching hospital) co-located with Flinders Private Hospital (FPH, private practice of EHO) by surgical trainees and the senior author (EHO) between July 2016 and October 2019. The T-14 questionnaire is completed by the parent/guardian and reports on the child’s symptoms and health-related QOL [5,6]. It is comprised of 14 Likert-scale questions, with a total score calculated (range 0–70), where a higher score signifies greater symptoms and lower health-related QOL. It is routinely administered by clinic staff to the parents of patients at the preoperative ENT (ear, nose, and throat) clinic appointment and at the first postoperative (approximately 6 weeks) clinic appointment, and filed in the patient’s medical chart. Six weeks is the standard time frame for postoperative review as surgical recovery is assessed as completed. The validation of the T-14 questionnaire was published by our group using this same time frame and methodology [4]. At the post-operative visit, parents/guardians were also questioned regarding visits to primary care providers and complications that did not result in hospital attendance, and this was documented in their medical chart. Patients were not specifically questioned about analgesia use unless they required medical review during their recovery. Patients are routinely discharged post-tonsillectomy with regular paracetamol and ibuprofen at FMC and FPH.

Inclusion criteria: Pediatric patients (16 years and under) who underwent a Bizact^TM^ tonsillectomy was included. The criteria for offering surgery in pediatric patients are sleep-disordered breathing (SDB) and recurrent tonsillitis (RT). SDB is based on the parental history of characteristic symptoms, including noisy breathing, disturbed sleep, snoring, and witnessed apneas [7]. The recurrent tonsillitis (RT) criteria for the surgery is based upon the Paradise criteria [8]. Exclusion criteria: patients with incomplete T-14 data or who underwent tonsillotomy or tonsillectomy by any other method (i.e., cold steel, coblation) were excluded for analysis for this study.

Surgical technique: The Bizact^TM^ device is manufactured by Medtronic (Minneapolis, MN, USA) and was approved for use by the Therapeutic Goods Administration in 2016. Tonsillectomy was performed using an extra-capsular dissection technique, as described by Krishnan et al. [1].

Data were collected from the patient medical chart after they had undergone Bizact tonsillectomy, completed follow up reviews, and included the following: demographic data (age, gender), indication for tonsillectomy (RT, SDB, RT, and SDB), operating surgeon grade (trainee, consultant) and postoperative complications (hemorrhage, readmissions). Post-tonsillectomy hemorrhage was graded using the Stammberger classification [9].

### 2.3. Statistical Methods

Data distribution was assessed for normality and found to be not normally distributed, therefore, non-parametric statistical tests were utilized (IBM SPSS Statistics for Windows, IBM Corp, Sydney, Australia. Released 2017). Data are presented as the median and interquartile range (IQR) or counts and percentages. The T-14 scores were compared preoperatively vs. 6-week postoperatively using related samples Wilcoxon Signed Rank test. The factors potentially affecting the magnitude of improvement (determined as a preoperative score–postoperative score) were analysed with a Spearman’s rho Correlation. The factors associated with secondary hemorrhage were analysed with a Spearman rho correlation.

## 3. Results

### 3.1. Patient Demographics

Patient demographic data are presented in Table 1. There were 146 Bizact^TM^ pediatric tonsillectomies performed. The most common indication for pediatric Bizact^TM^ tonsillectomy was SDB followed by RT and SDB.

### 3.2. T-14 Scoring

**Figure 1:**
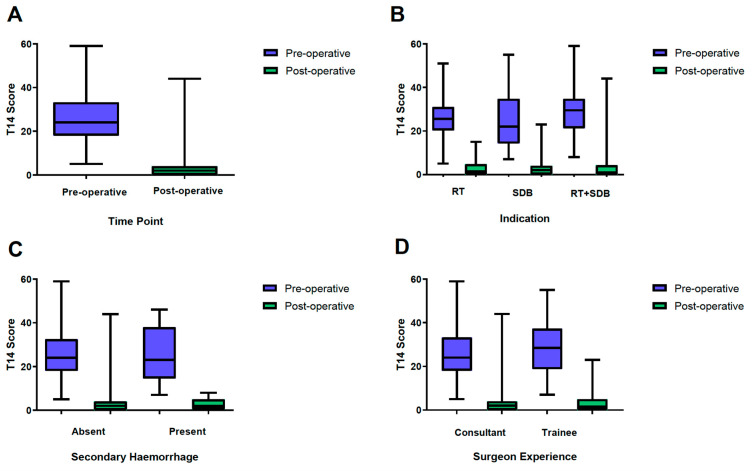
Parents reported quality of life following Bizact tonsillectomy. (**A**)T-14 scores demonstrate significant decrease from preoperative to 6 weeks postoperative; *p* < 0.001; *n* = 146. (**B**) Significant decrease in preoperative versus postoperative T-14 scores for presenting indication of recurrent tonsillitis (RT; *n* = 28; *p* < 0.001), sleep-disordered breathing (SDB; *n* = 81; *p* < 0.0001) and both recurrent tonsillitis with sleep-disordered breathing (RT + SDB; *n* = 24; *p* < 0.001 Related Samples Wilcoxon Signed Rank Test). A similar level of improved quality of life was observed across tonsillectomy indications (*p* > 0.05 Independent Samples Kruskal-Wallis). (**C**) Significant decrease in preoperative versus postoperative T-14 scores for those children that did or did not experience a post-tonsillectomy hemorrhage (PTH; absent *n* = 136; *p* < 0.0001; present *n* = 8; *p* = 0.012 Related Samples Wilcoxon Signed Rank Test). The presence of secondary hemorrhage did not impact on the improvement in quality of life following Bizact tonsillectomy (*p* > 0.05 Independent Samples Mann-Whitney U Test). (**D**) Significant decrease in preoperative versus postoperative T-14 scores when performed by Consultant or Trainee surgeon (consultant *n* = 108, *p* < 0.0001; trainee *n* = 38, *p* < 0.0001). Data presented as box-plots with whiskers representing minimum and maximum values.

There was a significant improvement in T-14 scores from a median of 24 (IQR 18–33) prior to surgery, to a median of 2 (IQR 0–4) at 6 weeks postoperatively (*p* < 0.001; Figure 1A). One patient had an increase in T-14 score (31 preoperatively to a score of 44 postoperatively), due to an episode of acute otitis media at the time of review. There was no significant difference in the magnitude of the improvement in the T-14 scores between the different indications for surgery (*p* > 0.05, Figure 1B), if there was a secondary post-tonsillectomy hemorrhage (PTH) (*p* > 0.05, Figure 1C), or between different surgeon experience levels (Figure 1D).

### 3.3. Surgeon Grade

Bizact^TM^ tonsillectomies were performed by a consultant otolaryngologist in 74.0% (108/146) and 7 trainees in 26.0% (38/146) of patients. Surgeons with a variety of experience levels were included in the study to determine the effectiveness of the technique with T-14 scores amongst varying surgeon experience levels. The results show that T-14 scores improve also when junior surgeons use the Bizact^TM^ device to perform tonsillectomy and the T-14 score change is not dependent on the experience of the surgeon (Figure 1D).

### 3.4. Post-Tonsillectomy Hemorrhage

There was no primary post-tonsillectomy hemorrhage. Secondary post-tonsillectomy hemorrhage was reported in 6.1% (9/146) of cases. Six were Stammberger grade A1/A2, and three were grade C. Post-tonsillectomy hemorrhage predominantly occurred between days 6 and 9. Three patients required return to theatre for cessation of bleeding resulting in a return to theatre rate of 2.1%. Age, gender, indication, or surgeon experience were not associated with secondary hemorrhage (all *p* > 0.05 Spearman’s rho).

### 3.5. Post-Operative Complications

Postoperative complications were captured by asking the parents of the patients at the postoperative review and via review of the patient’s encounters (visits) on Oacis (a statewide public hospital electronic system that captures any patient encounter at all public hospitals in South Australia). Complications requiring hospital admission due to dehydration, pain, or infection occurred in 2.1% (3/146) of cases occurring between postoperative Day 4 and Day 7. None of these patients developed post-tonsillectomy hemorrhage.

## 4. Discussion

This study investigates the change in T-14 scores in pediatric patients undergoing Bizact^TM^ tonsillectomy. We used the T-14 as a validated parental reported outcome measure for their child’s throat disorders [4]. It is important to assess patient-reported outcome measures when studying tonsillectomy outcomes besides the traditional outcomes such as hemorrhage, pain, other postoperative complications, and cost. Common tonsil related disorders such as sleep-disordered breathing (SDB) have the potential to result in developmental delay and behavioral difficulties if it is not treated appropriately [3]. Recurrent tonsillitis in a pediatric population can also result in days off school, frequent general practitioner visits, and a decreased quality of life compared to patients with juvenile arthritis and severe asthma [10]. These conditions are treated by tonsillectomy, therefore it is important to assess patient-reported outcome measures to determine the efficacy of the procedure. We used the T-14 as a validated parental reported outcome measure for their child’s throat disorders [2,4,5] and found that tonsillectomy using the new Bizact^TM^ device is associated with improved T-14 scores for pediatric patients.

Tonsillectomy by a variety of methods has been shown to improve short-term and long-term T-14 scores in various studies [2,4,5,7,11,12,13]. Lam et al. [4] investigated 63 pediatric patients who had undergone tonsillectomy by a variety of methods including 14 patients who had undergone a Bizact^TM^ tonsillectomy, 17 performed with coblation, 13 with cold steel, and 1 with monopolar. There was a significant decrease in the median T-14 score overall, from 23 (IQR 13–32) to 3 (IQR 0–7), but the improvement in T-14 score with each tonsillectomy method was not specified. None of the other studies assessing health-related quality of life outcomes based on T-14 scores have specified their method of tonsillectomy [2,4,5,7,11,12,13]. Dawe et al. found in 25 pediatric patients an improvement in T-14 scores from 45.6 (IQR 22.6–68.6) preoperatively to 7.2 (IQR 1.2–13.2) 6 months postoperatively [12]. Hopkins et al. reported the change in T-14 scores in 62 pediatric patients undergoing tonsillectomy, and 64 pediatric patients undergoing adenotonsillectomy. The 62 tonsillectomy patients had a mean preoperative T-14 score of 31.2 (95% confidence interval (CI) 28.9–33.5), and a mean change in T-14 score of 24.8 (95% CI 21.2–28.3) at 12–14 months postoperatively. The 64 adenotonsillectomy patients had a mean preoperative T-14 score of 34.9 (95% CI 32.5–37.4) with a mean change of 25.7 (95% CI 21.4–30.1). Neither the method of tonsillectomy nor the presence of complications was specified [5]. Konieczy et al. had 44 pediatric patients with T-14 scores recorded preoperatively, 3, 6, 12 and 24 months post-operatively. The T-14 scores improved from a mean of 33.3 (95% CI 29.7–36.9) preoperatively, to 3.4 (95% CI 1.8–5.0) at 3 months postoperatively, and this improvement was sustained at the 6, 12 and 24 month intervals. The current study determined that favourable changes in T-14 scores were obtained with pediatric patients undergoing Bizact^TM^ tonsillectomy with a median T-14 score of 24 (IQR 18–33) prior to surgery decreasing to 2 (IQR 0–4) 6 weeks postoperatively.

This study demonstrates the improvements in T-14 scores with Bizact^TM^ tonsillectomy occurred independently of indications for surgery, surgeon experience (trainee and consultant), and occurrence of post-tonsillectomy hemorrhage. Importantly, parents still reported significant improvement in their child’s T-14 score post-tonsillectomy in those children who experienced postoperative complications such as readmission or post-tonsillectomy hemorrhage. Furthermore, 38 of the tonsillectomies were performed by a trainee surgeon with similar favorable T-14 results to experienced consultants. This demonstrates that surgeons of a variety of experience levels are able to use the Bizact^TM^ device to treat tonsil-related diseases that impact upon health-related QOL. Compared with similar studies in the literature comparing T-14 scores, this study has the largest sample size. Additionally, no other study to date has analyzed whether health-related QOL outcomes, measured by T-14 scores, are affected by postoperative complications or surgeon experience level. [2,4,5,7,11,12,13]. The current study’s findings demonstrate that Bizact^TM^ tonsillectomy benefits a child’s QOL.

The Bizact^TM^ device for tonsillectomy is considered a “hot” technique, meaning that thermal energy is delivered to the tonsil bed during the procedure. Hot techniques are often reported to have higher secondary post-tonsillectomy hemorrhage rates (3.6–16.2%) than cold steel dissection (1–8.8%) and surgical ties or bipolar cautery for hemostasis [9,14,15,16,17]. Despite the potential for an increased secondary post-tonsillectomy hemorrhage rate, there are advantages to hot techniques over cold techniques, particularly in terms of reduced intraoperative blood loss and reduced operative time [18]. Primary hemorrhage rates are similar among each technique (0.5–1%) [9,14,15,16,17]. The current study found zero primary hemorrhages and a secondary post-tonsillectomy hemorrhage rate of 6.1%. The majority of post-tonsillectomy hemorrhages in this data set were minor Stammberger grade A bleeds, which did not require active intervention. The rate of secondary hemorrhage was higher in this study compared to the study by Krishnan et al. [1]. The reason for this remains unclear, but it is notable that their patient population was different from the current study given the inclusion of adult patients. Additionally, Krishnan’s study included only two senior consultant surgeons, whilst the current study included small numbers of junior trainees. It is possible that the inclusion of surgeons with lower experience levels resulted in a higher PTH rate, however, there were insufficient numbers of junior surgeons to conduct statistical analysis on the effect of PTH. This requires further research with a larger cohort of patients and different surgeon experience levels. The indication for tonsillectomy did not increase the likelihood of secondary hemorrhage. This contrasts with previous reports, where it is thought that the surgical indication of RT increases the probability of post-tonsillectomy hemorrhage [9]. In this cohort, 2.1% of patients (3/146) reported postoperative complications, two of which were related to decreased oral intake secondary to pain. It was reported that patients with increased postoperative pain are more likely to develop secondary hemorrhage [9] but this was not reflected in this population.

Regarding the cost, the Bizact^TM^ device is AUD 275 per unit, it costs less than the Coblation EVAC 70 wand, priced at AUD 375. It is more expensive than the monopolar diathermy wand and cold steel methods, which cost less than AUD 55 per patient. Despite being more expensive than some other techniques, the Bizact^TM^ has the potential to dramatically reduce operative time, minimizing time spent under a general anesthetic and maximizing theatre utilization. The significant improvement in T-14 scores following Bizact^TM^ tonsillectomy is important to know when using a new device for tonsillectomy. We surmise the tonsillectomy procedure itself, addressing the underlying medical condition (SDB and/or RT) is the primary reason for the benefit in the child’s quality of life and not a result of the Bizact^TM^ device itself.

There are a number of limitations in this study. The study design was to compare pre and postoperative T-14 scores following a Bizact^TM^ tonsillectomy, with each patient serving as their own control, rather than comparing different tonsillectomy techniques. We recommend that future studies could include a randomized control trial of Bizact^TM^ versus another tonsillectomy technique, with the T-14 questionnaire as one of the outcome measures. Postoperative pain is a common finding in tonsillectomy, and different techniques may result in varying levels of postoperative pain, potentially being severe enough to affect QOL or admission to hospital. Patients were routinely discharged on regular paracetamol and ibuprofen, but some patients were given oxycodone on discharge as determined by individual postoperative analgesia requirements. This study did not collect data regarding postoperative pain and this could be addressed in future studies. However, postoperative pain scores following Bizact^TM^ tonsillectomy were published [1]. Low numbers of surgical trainees included in this study, and their individual results were not sufficient for a comparison between consultant vs. trainee post-tonsillectomy hemorrhage rate to be reliably assessed. The Bizact^TM^ device requires the use of an extra-capsular technique and is not currently used for intracapsular tonsillectomy (tonsillotomy). In some cases, tonsillotomy is advantageous over tonsillectomy, as it results in less postoperative hemorrhage and pain, whilst still resulting in improved parent-reported QOL scores [19,20]. However, tonsillotomy was reported as having a 2.4% revision rate, [19] and parents may continue to opt for a tonsillectomy to avoid further surgical intervention in their child.

## 5. Conclusions

A Bizact^TM^ tonsillectomy in this study demonstrated an improvement in postoperative T-14 scores. The improvement in health-related QOL occurred in the majority of patients, irrespective of the occurrence of post-tonsillectomy complications or surgeon experience level. Further research of its benefits, outcomes, and safety with randomized control trials, and long-term quality of life data is recommended.

## Figures and Tables

**Table 1:** Patient Demographics.

Demographics	*N* = 146
Male: Female (number)	76:70
Age range (years)	1–16
Median age (years)	5.5
**Indication**	
Sleep Disordered breathing (SDB)(number)	84
Recurrent Infection (RT) (number)	28
SDB and Infection (SDB and RT) (number)	34

## Data Availability

The data presented in this study are available on request from the corresponding author. The data are not publicly available due to plan for further publication using this data bank.
